# *Interleukin‐1B* 31 C>T polymorphism combined with *Helicobacter pylori*‐modified gastric cancer susceptibility: evidence from 37 studies

**DOI:** 10.1111/jcmm.12737

**Published:** 2016-01-25

**Authors:** Hua‐Yong Ying, Bei‐Wei Yu, Zong Yang, Shan‐Shan Yang, Li‐Hong Bo, Xiao‐Yun Shan, Hui‐Jiao Wang, Yi‐Jun Zhu, Xue‐Song Wu

**Affiliations:** ^1^ Department of Laboratory MedicineJinhua Central HospitalJinhuaZhejiangChina; ^2^ Department of Laboratory MedicineJinhua People's HospitalJinhuaZhejiangChina; ^3^ The Fifth Medical TeamXinjiang Uygur Autonomous Region Corps of Chinese People's Armed Police ForcesXinjiangChina; ^4^ Department of Laboratory MedicineJinhua Woman & Children Health HospitalJinhuaZhejiangChina; ^5^ School of Humanities and Social ScienceHarbin Medical UniversityHarbinHeilongjiangChina

**Keywords:** interleukin‐1β, polymorphism, gastric cancer, meta‐analysis

## Abstract

Gastric cancer is one of the most common malignancies worldwide. Interleukin‐1‐beta (IL‐1β) is a pro‐inflammatory cytokine and potent inhibitor of gastric acid secretion. Some studies provided evidence of the association between *IL‐1B* 31 polymorphism and gastric cancer risk while other studies did not. Therefore, we conducted a comprehensive meta‐analysis to reassess the association. A systematic literature search of the PubMed and EMBASE databases identified 37 studies with 6108 cases and 8980 controls for this meta‐analysis. The crude odd ratios (ORs) and the 95% confidence intervals (CIs) were calculated to evaluate the strength of the association. Meta‐regression was used to determine the major source of heterogeneity across the studies. The pooled analysis did not suggest the significant association of *IL‐1B* 31 C>T polymorphism with gastric cancer risk. Stratified analysis was performed by ethnicity, source of control, genotype method, and indicated a significantly increased gastric cancer risk associated with *IL‐1B* 31T variant in the population‐based subgroup (heterozygous model: OR = 1.22, 95% CI = 1.03–1.45). Moreover, stratified analysis by *Helicobacter pylori* infection status indicated that *IL‐1B* 31 polymorphism increased gastric cancer risk in infection‐positive subgroup (homozygous model: OR = 1.35, 95% CI = 1.02–1.78; heterozygous model: OR = 1.31, 95% CI = 1.04–1.66; recessive model: OR = 1.29, 95% CI = 1.04–1.61). The study suggested that *IL‐1B* 31 polymorphism might confer susceptibility to gastric cancer in the presence of *H. pylori* infection, indicating a gene–environment interaction in gastric carcinogenesis.

## Introduction

Gastric cancer ranks as the fifth most frequently diagnosed malignancy and the second most common cause of cancer‐related death worldwide. *Helicobacter pylori* infection is a well‐establish risk factor for gastric cancer. However, the infection is considered a necessary but not sufficient cause of gastric adenocarcinoma, as the majority (80%) of cancer‐free individuals infected by *H. pylori* are asymptomatic 1, some may end up with duodenal ulcer disease and only a small fraction of them (<3%) ultimately develop gastric cancer in the lifetime 2. The reasons for the interindividual variability in *H. pylori*‐related clinical outcome remain unclear. Accumulating data indicate that gastric cancer is a complex multistep and multifactorial process, as well as result of gene–environment interactions. Genetic variations in some key genes that modify host's ability to response to environmental stimulation may help to explain the interindividual variability in the clinical outcome related to *H. pylori*.

Interleukin‐1‐beta (IL‐1β, encoded by IL‐1B) is a pro‐inflammatory cytokine 3 and also a potent inhibitor of gastric acid secretion 4, 5. Over the past decades, this cytokine has been implicated in gastric cancer carcinogenesis. There are three associated genes (*IL‐1A, IL‐1B* and *IL‐1RN*) on chromosome 2q, constituting an *IL‐1* gene cluster. The three genes correspond to three pro‐inflammatory cytokines IL‐1α, IL‐1β and IL‐1ra respectively. Among them, IL‐1β has been of particular interest because it closely influences the gastric physiological behaviour in response to *H. pylori* infection 3, 4, 5. When *H. pylori* invades stomach, IL‐1β production is up‐regulated to favour the initiation and amplification of the inflammatory response to the infection 3. Moreover, IL‐1β can decrease acidity of the stomach by effectively suppressing gastric acid secretion 4, 5. Thus far, three diallelic single nucleotide polymorphisms (SNPs) in the *IL‐1B* gene have been extensively studied, which occur at positions −511, −31 and +3954 base pairs (bps) upstream from the transcription start site 6. As the pooled analysis for the association of *IL‐1B* −511 and +3954 polymorphisms with cancer risk have been performed elsewhere in May 2013 7, we only focused on *IL‐1B* 31 polymorphism in the present study. The polymorphic *IL‐1B* 31 site is within the TATA box motif in the promoter region of this gene. Some evidence has shown that the *IL‐1B* promoters bearing T and C alleles have differential capacity of binding to nuclear proteins (*e.g*. transcription factors). As an example, lipopolysaccharide stimulation induced approximately a fivefold increase in the formation of DNA–protein complex in the presence of *IL‐1B* 31T oligonucleotide, while no effect on the *IL‐1B* 31C oligonucleotide was observed 6. Accordingly, *IL‐1B* 31C>T polymorphism has been shown to influence IL1β production, with variant T allele associated with enhanced expression of IL1β in comparison with wild‐type C allele 8, 9, 10.

Given the biological importance of *IL‐1B* 31 polymorphism*,* numerous studies have been carried out to explore its association with gastric cancer susceptibility. While some molecular epidemiology studies suggest that *IL‐1B* 31 genetic polymorphism is implicated in *H. pylori*‐related gastric carcinogenesis, others gain opposite results. Even two previous meta‐analyses conducted in 2010 and 2011 11, 12 also yielded contradictory results. Since then, many new studies on such association have been emerging. With this in mind, we conducted the updated meta‐analysis, aiming to provide a quality assessment of the association between *IL‐1B* 31 polymorphism and gastric cancer risk.

## Materials and methods

The latest meta‐analysis guidelines (PRISMA) were followed while carrying out the meta‐analysis, including literature search and data collection.

### Identification of the eligible studies

A comprehensive literature search of the PubMed and EMBASE databases was performed with the use of search terminology ‘IL‐1B’, ‘polymorphism or variation or variant’, ‘gastric or stomach’ and ‘cancer or carcinoma or tumour’. Literature search was started on October 15, 2014, and last updated on August 31, 2015 according to the latest meta‐analysis guidelines (PRISMA) 13. The reference lists of the selected original articles and reviews were manually screened to discover additional relevant studies.

### Inclusion and exclusion criteria

The studies included in the meta‐analysis had to meet the criteria: (*i*) either case–control study or cohort study; (*ii*) original study investigating the association of *IL‐1B* 31 polymorphism with gastric cancer susceptibility; (*iii*) adequate information to calculate odd ratios (ORs) and 95% confidence intervals (CIs). We generally ruled out study that exhibited significant departure from Hardy–Weinberg equilibrium (HWE) (*P*_*HWE*_ < 0.05) in control participants, except that another *IL‐1B* polymorphism passed HWE check in the same study. If the same participants appeared in multiple studies, only the latest or the largest study was chosen. Exclusion criteria were: (*i*) overlapped participants, (*ii*) abstract, case report, comment and review, (*iii*) insufficient genotyping data and (*iv*) studies with participants having family history of cancer, or family‐based studies.

### Data collection

Two authors separately extracted detailed information from all the eligible articles.

If there was controversy, it was resolved by full discussion between the two authors until they reached an agreement. Data extracted from eligible studies were as follows: name of the first author, year of publication, country in which studies were conducted, ethnicity, genotype counts of cases and controls, source of controls, genotyping method and the *P*‐value of HWE in controls. The stratified analysis was performed by ethnicity (Asians, Caucasians, Africans or Mixed which contained more than one ethnic group) and the source of controls (HB: hospital based and PB: population based), and genotype methods. In this meta‐analysis, the hospital‐based controls were not necessarily collected from individuals in hospital for gastric‐related pathologies in the same period of time. A study was considered as a population‐based case–control study, if cases were from a precisely defined population and controls were randomly chosen from the same population. In a hospital‐based case–control study, controls should be from a medical facility in which cases were recruited. As PCR‐restriction fragment length polymorphism (PCR‐RFLP) and PCR with confronting two‐pair primers (PCR‐CTPP) may not be as accurate as other methods (*e.g*. Taqman and sequencing), we categorized studies employing PCR‐RFLP/CTPP into one group, and rest of studies into the other groups. More than one study might be extracted from a article that contain multiple populations from different region 14 or studies with different design 15.

### Quality assessment

The Newcastle–Ottawa Scale is commonly used to appraise the quality of studies collected in a systematic review and/or meta‐analysis. Basically, each study is scored based on three aspects: the selection of the study groups (four quality items), the comparability of the groups (one item) and the ascertainment of outcome (three items).

Stars are granted for every quality item and studies of the highest quality are awarded a maximum of nine stars 16.

### Statistical methods

The crude ORs and 95% CIs were computed to measure the association between *IL‐1B* 31 polymorphism and gastric cancer risk. Four genetic models were used to calculated risk estimates: homozygous model (TT *versus* CC), heterozygous model (TC *versus* CC), dominant model (TT + TC *versus* CC) and recessive model (TT *versus* TC+CC). We used *Z* to determine the significance of an association. Cochran *Q*‐test and *I* ^2^ statistic were used to analyse the between‐study heterogeneity. If there was no heterogeneity, *i.e. P*‐value for *Q*‐test was equal to or larger than 0.10, fixed‐effect model was used; otherwise, a random‐effect model was chosen. Leave‐one‐out sensitivity analysis was conducted by sequentially eliminating one single study at a time and recalculating ORs and 95% CI. Chi‐squared test was used to check deviation from HWE in controls. Additionally, the symmetry of the funnel plot was assessed by Egger's liner regression test to detect the potential publication bias. Lastly, a meta‐regression analysis was carried out to determine the main sources of the heterogeneity in the present meta‐analysis. All statistical tests were performed by STATA version 11.0 (STATA Corporation, College Station, TX, USA). A two‐sided *P*‐value of <0.05 was considered statistically significant.

## Results

### Study characteristics

**Figure 1:**
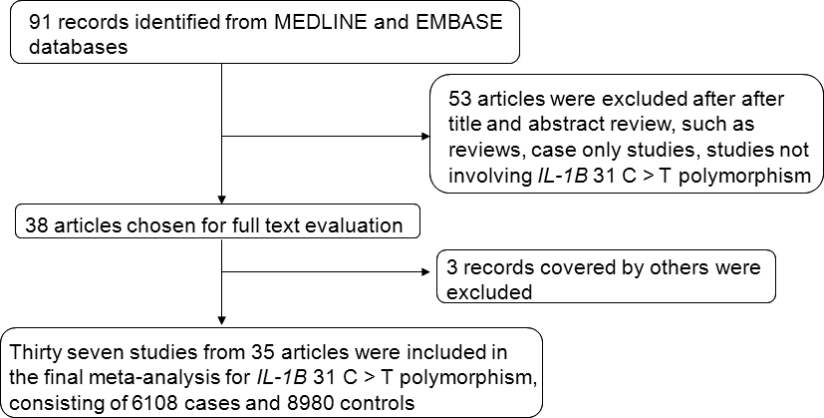
The flow chart of the identification of eligible articles.

A total of 91 pertinent articles were retrieved after the initial database search. We then checked the title and abstract of these publications for study eligibility. As a result, 53 articles were excluded as they failed to meet eligibility criteria, due to not investigating *IL‐1B* 31 polymorphism, or not case–control study. Of the remaining 38 publications, three were eliminated, due to reporting duplicate data 17, 18, 19. Ultimately, 37 studies from 35 publications 6, 10, 14, 15, 20, 21, 22, 23, 24, 25, 26, 27, 28, 29, 30, 31, 32, 33, 34, 35, 36, 37, 38, 39, 40, 41, 42, 43, 44, 45, 46, 47, 48, 49, 50 including 6108 cases and 8980 controls were included in the meta‐analysis (Fig. 1). Twenty studies were conducted among Caucasians, and 17 among Asians. In term of study design, 28 were HB studies, and nine were PB studies. PCR‐RFLP and PCR‐CTPP were performed in 21 studies, while the retained studies employed technologies including Taqman and sequencing. Among the selected publication, seven (1338 cases and 1875 controls) provided genotype counts of cases and controls by *H. pylori* infection status (Table 1). Additionally, one study only provided counts of CC and TT + TC genotypes, without information of either TT or TC genotype 31. Therefore, this study was only included while the dominant model was employed. All the cases were histologically confirmed in each of the included studies.

**Table 1:** Characteristics of the 37 studies for the association of IL‐1B 31 polymorphism and gastric cancer risk

First author	Year	Country	Race	Design	Genotyping method	Case	Control	MAF	HWE	Score
El‐Omar	2000	USA	Caucasian	PB	PCR‐SSCP/Taqman	366	429	0.7	0.070	9
Zambon	2002	Italy	Caucasian	HB	PCR‐RFLP	23	276	0.33	0.520	9
Zeng	2003	China	Asian	HB	PCR‐RFLP	84	192	0.9	0.517	8.5
Zeng	2003	China	Asian	HB	PCR‐RFLP	86	169	0.86	0.023	8.5
Wu	2003	Taiwan	Asian	HB	Sequencing	220	230	0.45	0.145	9
Lee SG	2003	Korea	Asian	HB	Sequencing	190	172	0.51	0.222	9
Gatti	2004	Brazil	Caucasian	HB	PCR‐CTPP	56	56	0.48	0.106	7.5
Glas	2004	Germany	Caucasian	HB	PCR‐RFLP	88	145	0.35	0.139	9
Yang	2004	China	Asian	PB	PCR‐RFLP	280	258	0.52	0.734	8.5
Palli	2005	Italy	Caucasian	PB	Taqman/RFLP	158	546	0.34	0.443	9
Garza‐Gonzalez	2005	Mexico	Caucasian	HB	PCR‐RFLP	63	215	0.56	0.278	8
Ruzzo	2005	Italy	Caucasian	HB	PCR‐RFLP	138	100	0.37	0.316	8
Chang	2005	Korea	Asian	HB	PCR‐RFLP	234	434	0.52	0.005	7
Lu	2005	China	Asian	PB	DHPLC	250	300	0.54	0.990	7.5
Rocha	2005	Brazil	Caucasian	HB	PCR‐CTPP	166	536	0.45	0.822	7.5
Tatemichi	2005	Japan	Asian	HB	PCR‐CTPP	156	176	0.26	NA	8
Zhang	2005	China	Asian	PB	PCR‐RFLP	154	166	0.43	0.576	7.5
Alpízar‐Alpízar	2005	Costa Rica	Caucasian	PB	PCR‐RFLP	50	50	0.59	0.728	7
Sicinschi	2006	Mexico	Caucasian	HB	Taqman	183	377	0.55	0.006	8
Sugimoto	2006	Japan	Asian	HB	PCR‐RFLP	105	172	0.5	0.445	8
Kamanger	2006	Filand	Caucasian	PB	Taqman	112	207	0.39	0.070	8.5
Ikehara	2006	Japan	Asian	HB	PCR‐CTPP	271	271	0.45	0.205	7.5
Al‐Moundhri	2006	Oman	Asian	HB	Taqman	118	245	0.59	0.000	7
Seno	2007	Japan	Asian	HB	Sequencing, *etc*.	99	92	0.45	0.446	7.5
Zhang	2007	China	Asian	HB	PCR‐RFLP	214	230	0.47	0.393	7.5
LEE KA	2007	Korea	Asian	HB	MALDI‐TOF	342	515	0.54	0.654	8
Sitarz	2008	Countries	Caucasian	HB	Taqman	241	100	0.41	0.281	8
Persson	2009	Sweden	Caucasian	PB	Pyrosequencing	284	242	0.34	0.727	9
Persson	2009	Sweden	Caucasian	HB	Pyrosequencing	65	294	0.38	0.680	9
Kumar	2009	India	Asian	HB	PCR‐RFLP	136	110	0.5	0.036	7.5
Wex	2010	Germany	Caucasian	HB	PCR‐RFLP	116	94	0.32	0.840	8.5
Li	2010	China	Asian	HB	PCR‐RFLP	140	165	0.39	0.000	8
He	2011	China	Asian	HB	PCR‐RFLP	392	508	0.54	0.155	8
López‐Carrillo	2012	Mexico	Caucasian	HB	TaqMan and Pyrosequencing	158	317	0.65	0.006	8
Qiu	2014	China	Asian	HB	MassARRAY	52	52	0.54	0.246	7.5
Gonzalez‐hormazabal	2014	Chile	Caucasian	HB	TaqMan	147	172	0.54	0.819	7.5
Wang	2014	China	Asian	PB	PCR‐RFLP	171	367	0.51	0.723	8

HB, Hospital based; PB, Population based; PCR‐SSCP, polymerase chain reaction single‐strand conformation polymorphism; PCR‐RFLP, PCR‐restriction fragment length polymorphism; PCR‐CTPP, PCR with confronting two‐pair primers; DHPLC, denaturing high‐performance liquid chromatography; MALDI‐TOF, Matrix‐assisted laser desorption‐ionization time‐of‐flight; MAF, Minor allele frequency; NA, Not applicable.

### Quantitative synthesis

**Figure 2:**
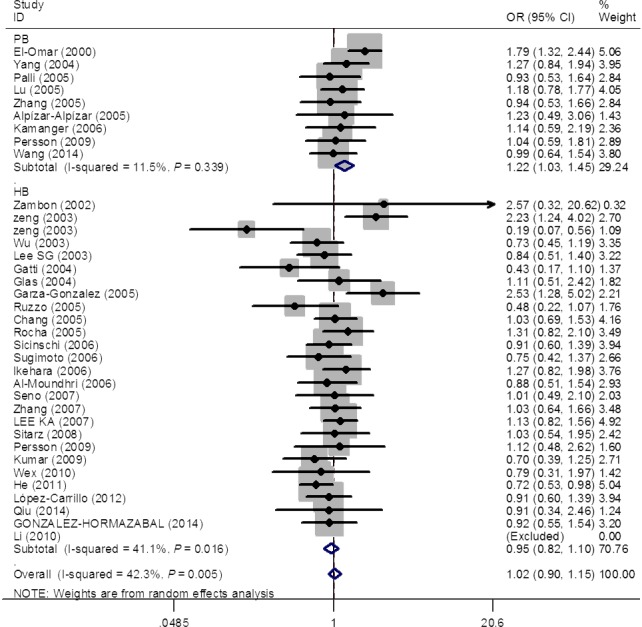
Forest plot for the risk of gastric cancer with *IL‐1B* 31 C>T polymorphism (heterozygous model). For each study, the estimates of OR and its 95% CI are plotted with a box and a horizontal line.

**Figure 3:**
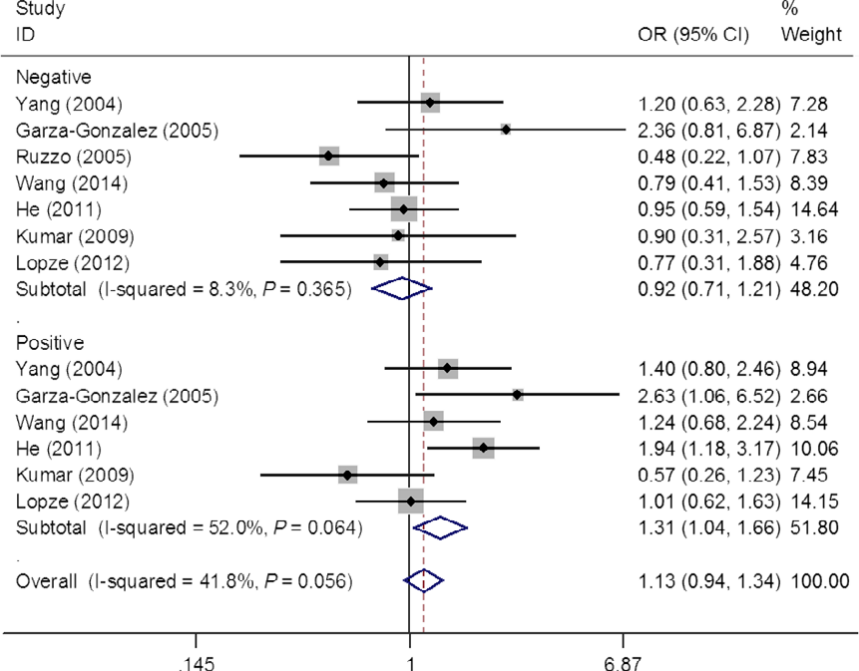
Forest plot for the risk of gastric cancer with *IL‐1B* 31 C>T polymorphism stratified by *Helicobacter pylori* infection status (heterozygous model).

Overall, this systematic review produced 37 eligible studies, consisting 6108 cases and 8980, for the pooled analysis of the association between *IL‐1B* 31 polymorphism and gastric cancer risk. Despite its biological plausibility, pooled risk estimates failed to provide an evidence of such association (homozygous model: OR = 1.02, 95% CI = 0.89–1.18; heterozygous model: OR = 1.02, 95% CI = 0.90–1.15; dominant model, OR = 1.03, 95% CI = 0.93–1.15 and recessive model: OR = 1.02, 95% CI = 0.91–1.15) (Fig. 2 and Table 2). Upon the removal of studies 10, 14, 34, 38, 43, 46, 50 deviated from HWE, risk estimates were not substantially changed (homozygous model: OR = 1.05, 95% CI = 0.89–1.24; heterozygous model: OR = 1.07, 95% CI = 0.94–1.22; dominant model, OR = 1.08, 95% CI = 0.95–1.23 and recessive model: OR = 1.04, 95% CI = 0.92–1.18). Stratified analysis by ethnicity and source of control observed significant association among PB‐based studies under the heterozygous model (OR = 1.22, 95% CI = 1.03–1.45; Fig. 2). The stratified analysis by genotype method did not yield significant result. Interestingly, seven studies reported genotype count for *H. pylori* infection status separately and allowed stratified analysis by infection status. Stratified analysis observed that significantly increased gastric cancer risk was associated with *IL‐1B* 31 polymorphism in *H. pylori*‐positive subgroup, while null association was observed in *H. pylori*‐positive subgroup (Fig. 3 and Table 2).

**Table 2:** Meta‐analysis of the association between *IL‐1B* 31 polymorphism and gastric cancer risk

Variables	No. of studies (cases/controls)	Homozygous			Heterozygous			Recessive			Dominant		
		TT *versus* CC			TC *versus* CC			TT *versus* (TC & CC)			(TT & TC) *versus* CC		
		OR (95% CI)	*P* het	*I* ^2^ (%)	OR (95% CI)	*P* het	*I* ^2^ (%)	OR (95% CI)	*P* het	*I* ^2^ (%)	OR (95% CI)	*P* het	*I* ^2^ (%)
All	37 (6108/8980)	1.02 (0.89–1.18)	0.004	43.2	1.02 (0.90–1.15)	0.005	42.3	1.03 (0.93–1.15)	0.015	37.1	1.02 (0.91–1.15)	0.001	48.3
Ethnicity													
Caucasian	17 (2414/4156)	1.07 (0.87–1.31)	0.121	29.6	1.08 (0.89–1.31)	0.037	41.7	1.04 (0.91–1.19)	0.237	18.5	1.08 (0.90–1.30)	0.036	41.8
Asian	20 (3694/4824)	1.0 (0.82–1.22)	0.005	52.4	0.97 (0.83–1.13)	0.038	40.6	1.03 (0.88–1.21)	0.007	49.9	0.99 (0.85–1.15)	0.005	51.2
Source of control													
HB	28 (4283/6415)	0.92 (0.80–1.06)	0.204	18.2	0.95 (0.83–1.10)	0.016	41.1	0.98 (0.87–1.11)	0.075	29.7	0.96 (0.84–1.09)	0.029	37.1
PB	9 (1825/2565)	1.27 (0.95–1.69)	0.018	56.9	**1.22 (1.03–1.45)**	0.339	11.5	1.14 (0.94–1.39)	0.055	47.4	1.22 (0.99–1.50)	0.067	45.3
*Helicobacter pylori* infection													
P (+)‐matched	6 (774/954)	**1.35 (1.02–1.78)**	0.000	79.7	**1.31 (1.04–1.66)**	0.064	51.8	**1.29 (1.04–1.61)**	0.008	68.1	0.99 (0.59–1.66)	0.001	75.2
N (−)‐matched	7 (564/921)	0.91 (0.66–1.25)	0.360	9.1	0.92 (0.71–1.21)	0.365	8.3	0.93 (0.72–1.20)	0.561	<0.001	1.01 (0.71–1.44)	0.159	35.3
Genotype method													
RFLP/CTPP	21 (3123/4690)	0.93 (0.74–1.16)	0.013	46.9	1.02 (0.90–1.15)	0.001	56.4	0.97 (0.83–1.13)	0.027	41.6	0.99 (0.82–1.19)	0.001	56.8
Others	16 (2985/4290)	1.12 (0.94–1.34)	0.105	32.1	1.06 (0.93–1.21)	0.328	11.0	1.10 (0.96–1.26)	0.146	27.6	1.07 (0.93–1.23)	0.114	31.1

Het, heterogeneity; HB, Hospital based; PB, Population based; FB, family based. The results were in bold, if the 95% CI excluded 1 or *P* < 0.05.

There was evidence of significant between‐study heterogeneity among overall studies of the *IL‐1B* 31 polymorphism and gastric cancer risk under all the genetic models. Furthermore, the inclusion of seven studies deviated from HWE did not qualitatively affected between‐study heterogeneity. Moreover, stratified analysis by genotype indicated significant between‐study heterogeneity in PCR‐RFLP/CTPP group rather than in the other group. This result suggested that genotype method might be a source of heterogeneity. We next conducted meta‐regression to explore whether ethnicity and source of control would account for the heterogeneity across the studies. As indicated in Table 3, source of control seemed to be responsible for the heterogeneity in the meta‐analysis (homozygous model: *P* = 0.036), but not ethnicity (homozygous model: *P* = 0.762; heterozygous model: *P* = 0.417); dominant model: *P* = 0.537; recessive model: *P* = 0.949).

**Table 3:** Meta‐regression analysis of the main characteristics of the 37 studies

Study characteristics	Homozygous			Heterozygous			Recessive			Dominant		
	Coef.	95% CI	*P*	Coef.	95% CI	*P*	Coef.	95% CI	*P*	Coef.	95% CI	*P*
Ethnicity	0.042	(−0.24, 0.32)	0.762	0.10	(−0.15, 0.35)	0.417	−0.007	(−0.23, 0.22)	0.949	0.07	(−0.16, 0.31)	0.537
Source of controls	0.321	(0.02, 0.62)	**0.036**	0.21	(−0.05, 0.48)	0.115	0.154	(−0.09, 0.39)	0.203	0.23	(−0.02, 0.49)	0.068

The results were in bold, if the 95% CI excluded 1 or *P* < 0.05.

### Sensitivity analysis

Leave‐one‐out sensitivity analysis was used to measure the stability and reliability of the present meta‐analysis. Briefly, a study was removed from pooled data one at a time, followed by the recalculation of ORs and 95% CIs. Consequently, no omitting of a single study changed the result substantially.

### Publication bias

**Figure 4:**
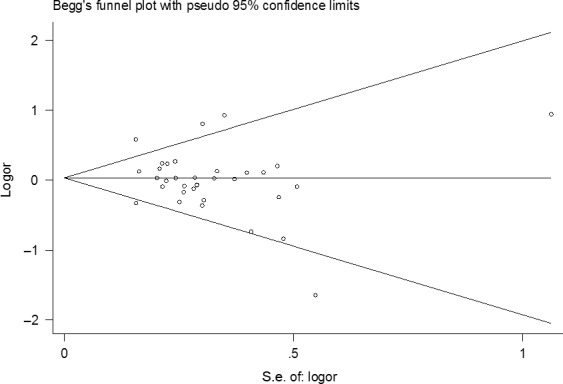
Begg's funnel plot for publication bias test (heterozygous model). Each point represents a separate study for the given association.

Begg's funnel plot was used to examine the potential publication bias in the meta‐analysis. The symmetrical funnel plots suggested no publication bias for the association between *IL‐1B* 31 polymorphism and overall gastric cancer risk under all the genetic models (Fig. 4). The lack of publication bias was further substantiated by Egger's weighted regression test (homozygous: *P* = 0.881; heterozygous, *P* = 0.203; dominant, *P* = 0.398; recessive *P* = 0.619).

## Discussion

*Helicobacter pylori* infection is considered as a leading cause of gastric cancer, with half of the world's population infected by this type of bacterium. *H. pylori* infection triggers the release of a panel of pro‐inflammatory cytokines in human gastric epithelial cells, including IL‐1β, which plays a key role in the initiation and amplification of the inflammatory response to this infection 51, 52, 53. Besides its indispensible role in the inflammation, IL‐1β is also recognized as a strong inhibitor of gastric acid secretion 4, 5. Given its functional importance, potential functional polymorphisms in the *IL‐1B* gene that may influence IL‐1β production have drawn great attention for their association with gastric cancer risk. In this meta‐analysis of 37 studies including 6000 cases and 8483 controls, we did not provide evidence of the association between *IL‐1B* 31 polymorphism and gastric cancer. Alternatively, stratified analysis by infection status observed significant association among *H. pylori* infection‐positive subgroups. Two meta‐analyses were performed for the association of *IL‐1B* 31 polymorphism and gastric cancer risk. One was performed by Xue *et al*. in 2010 11, in which 21 studies with 3786 cases and 5883 controls were included, and no association was found between *IL‐1B* 31 polymorphism and gastric cancer risk. The other one by He *et al*. in 2011 12 investigated the association between this variant and overall cancer risk, in which 25 studies with 4392 case and 6819 control involved gastric cancer risk. This study yielded a significant association between the variant of interest and gastric cancer risk. However, as this meta‐analysis covered overall various cancer types, the association of *IL‐1B* 31 polymorphism and gastric cancer risk were not analysed deeply enough. For example, stratified analysis and the source of between‐study heterogeneity were not explored for the gastric cancer risk. Moreover, neither of the two meta‐analyses considered the effect of *H. pylori* infection on the association of interest. The overall null association in the present meta‐analysis was consistent with the former meta‐analysis 11. A total of 35 articles with cases and controls were pooled together in the current meta‐analysis. Two articles 17, 19 included in the meta‐analysis by He *et al*. were not selected for our study because of overlapped participants. Finally, a total of 12 new studies were supplemented to our meta‐analysis, when compared to the latter one. Moreover, we extracted two studies from either Persson's or Zeng's study, as Persson's study consisted of one HB and one PB study and Zeng's study contained two studies involving participants recruited from different region. Genetic variation may modulate individuals' responses to cancer‐related infection. He *et al*. had attempted to examine gene–environment (virus) interaction in cancer susceptibility, by combing three studies related to gastric cancer infected with *H. pylori* and four related to hepatocellular cancer infected with hepatitis C or hepatitis B virus 12. They found that the risk effects of variant allele of *IL‐1B* 31T on gastric cancer were more pronounced in *H. pylori* infection‐positive subgroup than in negative group 12. Unlike their meta‐analysis involving both *H. pylori* and hepatitis C or hepatitis B virus, to the best of our knowledge, the current one is the first meta‐analysis to explore how *H. pylori* infection modified the association of *IL‐1B* 31 polymorphism and gastric cancer risk. Although no association was found between *IL‐1B* 31 polymorphism and overall gastric cancer, this variant was shown to significantly increase gastric cancer risk in *H. pylori* infection‐positive subgroup, but not in negative group. Our findings are in lines with some previous studies 11, 27, 45.

Being a low penetrant SNP, *IL‐1B* 31 polymorphism might contribute to gastric cancer susceptibility; however, such risk effect seemed to be dependent on other risk factors, such as *H. pylori* infection, suggesting gene–environment interaction in gastric carcinogenesis. *H. pylori* infection is associated with increased gastric epithelial damage, and may lead to different clinical outcome including gastric cancer and duodenal ulcer disease 54. The mechanisms by which *H. pylori* incites gastric carcinogenesis remain unclear. Its infection usually triggers inflammatory response in the gastric mucosa, with few exceptions. Some evidence has suggested that the crucial role of *H. pylori* in carcinogenesis is associated with its effects on gastric acid secretion. For instance, *H. pylori*‐induced gastritis that was restricted to the antral region usually led to inordinate acid secretion and was consequently prone to duodenal ulcer disease 55. On the contrary, some infected individuals with gastritis affecting the acid‐secreting corpus region might develop extensive corpus gastritis and further progressed to hypochlorhydria and gastric atrophy 56, and thereby had increased risk of gastric cancer 57, 58. It is well‐known that IL‐1β is not only an important pro‐inflammatory cytokine 3 but also a potent inhibitor of gastric acid secretion 4, 5, 59. Chang *et al*. studied 434 controls and 234 patients with GC and demonstrated that carrier of *IL‐1B* 31TT genotype had significantly higher mucosal IL‐1β levels than those carrying *IL‐1* 31TC or *IL‐1B* 31CC genotype among *H. pylori*‐infected Korean GC patients 10. Taken together, it is biological plausible that *H. pylori* may combined with *IL‐1B* 31T to confer increased susceptibility to gastric cancer.

Despite these interesting findings, attention should be paid to some limitations in the current up‐to‐date meta‐analyses. First, although the number of cases and controls in the pooled analysis was moderate, the sample sizes of single studies were relative smalls, which might be attributable to the heterogeneity in this meta‐analysis. Second, as most studies did not show detailed genotype counts according to tumour stages and grades, we were unable to evaluate the association between *IL‐1B* 31 polymorphism and gastric cancer in the stratified analysis by tumour stages and grades. Because of the same reason, the missing data of cancer stage and insufficient histological details may limit us to further explore the effect of *H. pylori* on gastric cancer. Third, crude ORs were used to determine the association, because usually only aggregate data were presented in research articles, *i.e*. genotype counts were not reported separately by sex, age, smoking status, tumour stages and grades. We were not able to adjust for potential confounders. Therefore, these results should be interpreted carefully. Finally, the source of controls was found to be a significant cause of heterogeneity only in the homozygous model. One possible reason could be that the percentage of variant homozygotes varied greatly among the included studies.

In summary, we did not replicate the reported association between and gastric cancer susceptibility. Nevertheless, we substantiated that *IL‐1B* 31T conferred genetic susceptibility to gastric cancer among *H. pylori* infection‐positive individuals.

Our results underscore the significance of gene–environment interactions in determining the gastric cancer susceptibility. However, these findings require further validation by large, well‐designed case–control studies involving different ethnicity.

## Conflicts of interest

None.
